# Micro-CT and histological examination of accessory canals in 34 equine cheek teeth

**DOI:** 10.3389/fvets.2024.1396871

**Published:** 2024-04-10

**Authors:** Szabolcs A. Korsós, Carsten Staszyk, Matthieu Boone, Iván Josipovic, Jörg Vogelsberg, Lieven Vlaminck

**Affiliations:** ^1^Department of Large Animal Surgery, Anaesthesia and Orthopaedics, Faculty of Veterinary Medicine, Ghent University, Ghent, Belgium; ^2^Institute of Veterinary-Anatomy,-Histology and -Embryology, Faculty of Veterinary Medicine, Justus-Liebig-University Giessen, Giessen, Germany; ^3^Department of Physics and Astronomy – Radiation Physics, Faculty of Science, Radiation Physics Research Group – Centre for X-ray Tomography of the UGent, Ghent University, Ghent, Belgium

**Keywords:** equine dentistry, accessory canals, apical delta, endodontics, horse

## Abstract

Accessory canals and apical deltas have been extensively studied in human dentistry. Their clinical role as a difficult to clean reservoir for bacteria during endodontic treatments has been well described. Many papers describe in detail the pulp anatomy of equine dentition but little attention has been given to their apical ramifications. The goal of this paper is to describe the presence and anatomy of these accessory canals and apical deltas in healthy equine cheek teeth and discuss their possible relevance in the light of equine endodontics. To accomplish this, 15 maxillary and 19 mandibular healthy cheek teeth were collected ranging from Triadan 06 s to 11 s with eruption ages from 4 to 9 years. Their root anatomy was documented in detail based on micro computed tomography images. A subset of 3 teeth also underwent histological examination. Accessory canals were found in all but two teeth examined. Up to 44 accessory canals per tooth have been found with locations ranging from the root furcation down to the apex of the root and with highly variable diameters. Apical deltas in different stages of development were found in 84% of the roots. The presence of accessory canals identified on microCT images could be confirmed using histological examination although some of them were obliterated by reparative dentin. Accessory canals can be found in most equine cheek teeth and add complexity to their endodontic anatomy. This could have important implications for their treatment in case of pulp pathology. In humans, failure to remove bacterial biofilm from such canals has been associated with failure of endodontic treatment. Research on diseased equine teeth is required to gain a better understanding of their clinical relevance in horses.

## 1 Introduction

Many authors have described in detail the dynamic anatomy of the pulp system in equine cheek teeth and incisors. Studies conducted with the use of dental sectioning (1–5), histology (1–3, 5–8), radiography (1, 5, 6, 9), (micro) computed tomography imaging (CT) (3–7, 10, 11) and Magnetic Resonance Imaging (MRI) (9) revealed an intricate, ever-changing system of interconnected pulp horns, pulp canals and pulp chambers (12, 13). However, besides a brief mention (7), none of these papers focused on details of the apex of equine cheek teeth in form of an apical delta formation and/or accessory root canals.

Starting from the occlusal surface, the 5 to 7 pulp horns (3) are protected by a layer of a special kind of secondary dentine, referred to as irregular secondary dentine, with a thickness that varies between 2 and 33 mm (3, 14). In cheek teeth up to an eruption age of 6 years these pulp horns can originate from a common pulp chamber at the deeper level of the reserve crown just coronally to the bifurcation/ trifurcation. Finally, the pulp canals communicate with the periapical tissues through apical foramina at the tip of each root.

With increasing age, the continuous physiological deposition of secondary dentine will result in several changes of the endodontic anatomy. Pulp horns and root canals get progressively narrower and the common pulp chamber divides into several compartments. The root canals of mandibular cheek teeth and the palatal root of maxillary cheek teeth have been observed to split up into two to three root canals toward the apex (4). As dentine deposition continues, apical foramina size becomes macroscopically invisible (2).

Gasse et al. (2) were the first to note that vascularized, vital pulp tissue is still present in equine cheek teeth without a macroscopically visible apical foramen. Histology of the apical region of these teeth revealed microscopic canals filled with blood vessels and connective tissue connected to the main pulp canal. Although actual communication with periapical tissues could not be identified in this study it was suggested that microscopic vascular communications between the pulp and the periodontium would exist in order to keep the pulp alive. Similar microscopic canals have been well studied and described in human dentistry (15–19). They are called accessory canals and have been defined as “any branch of the main pulp canal or chamber that communicates with the external surface of the root” (20). When the root canal divides into several of such accessory canals at or near the apex, this arrangement of canals is called an apical delta (20). It has been suggested that accessory canals are formed during the secondary dentine formation by entrapment of periodontal blood vessels and connective tissue in Hertwig’s epithelial root sheath (21). Their physiological role might be additional blood supply to the pulp tissue.

They have also been described in other brachydont species such as dogs (22–26) and cats (27, 28). Additionally, even hypsodont cheek teeth of alpacas (29) and equine incisors can have accessory canals.

During endodontic treatment accessory canals present hard to instrument zones which provide a safe haven for bacteria and inflamed tissues (30, 31). In humans they are known to be a possible source of post treatment root infections. Arnold et al. (32) have shown the presence of a biofilm inside these canals after endodontic debridement and disinfection. Possible solutions to address this problem, include instrumentation when accessible, apex resection, using (ultrasonic) agitation techniques to maximize the power and spread of irrigants, the use of a two-step procedure with pulp medication and finally extraction of the tooth (30, 33). Filling/sealing of accessory canals has also been proposed as a possible therapy but has been subject to a lot of controversy (31). Good knowledge of the existence and spatial anatomy of accessory canals in equine teeth is imperative in the light of designing more effective endodontic protocols and applying endodontic therapy as a treatment for pulpitis and/or apical disease in horses. The aim of this study is to describe the detailed anatomy (direction, size, branching pattern) of accessory canals in equine cheek teeth using micro computed tomography imaging.

## 2 Materials and methods

### 2.1 Sample collection and storage

Equine cheek teeth were collected from fresh cadaver heads of 10 warmblood horses from the slaughterhouse, and from cadaver heads of horses that were euthanized for reasons unrelated to any dental pathology. For the use of this second group of specimens owner’s consent was collected on admittance of the horses to our clinics. Only permanent cheek teeth that did not show any signs of (previous) infection or malformation and could be extracted without damage to the roots were included in this study. The teeth were individually stored in plastic containers filled with 0.5% chloramine solution at ambient temperature until further processing. The roots of the teeth were embedded in polymethyl methacrylate (PMMA) for easy positioning during micro computed tomography processing.

The age of the horse cadavers used in this study was estimated based on the occlusal morphology of the incisors (34). In this study we refer to the “dental age” or “eruption age” which is the number of years since the eruption of the tooth in the oral cavity.

### 2.2 Micro CT imaging

High-resolution X-ray CT (microCT) imaging was performed on all collected cheek teeth. Each tooth was scanned individually in the custom–built scanner system HECTOR (35) using 150kVp tube voltage and target power of 25 W. Using a geometrical magnification of 4.92, a reconstructed voxel size of 40.3 μm was achieved. Per sample, 2001 projection images of 1,000 ms integration time were acquired over a full 360° rotation. The projection data was reconstructed to a 3D volume using the in-house developed software package Octopus Reconstruction (36). A commercial 3D rendering software package (VGStudioMAX, Volume Graphics GmbH, Germany) was used to determine different parameters including number of accessory canals, canal types, presence of an apical delta, accessory canal location, direction, smallest diameter and patency.

The accessory canal types and apical delta definition were based on the AAE glossary of endodontic terms (20). The nomenclature of the different types of accessory canals (furcation canal, recurring canal) were adapted from De Deus (37).

Due to the lack of an unambiguous definition of when a canal belongs to an apical delta and when it is a “regular accessory canal” the decision was made to not make this differentiation in this paper and simply call every branch of the root canal an accessory canal. Although the presence of an apical delta was noted, the number of accessory canals that make up the apical delta was not. The location of an accessory canal was determined based on the location of its accessory apical foramen.

Accessory canal direction was determined by comparing the relative position of the pulpal and peripheral end of an accessory canal. More specifically the level where the accessory canal splits off of the root canal and penetrates the inner surface of the root, will be termed “internal accessory foramen.” Vice versa, the position where the accessory canal penetrates the peripheral surface of the root toward the periapical tissues will be termed “accessory apical foramen.” If the accessory apical foramen was located more occlusally than the internal accessory foramen, the direction of this accessory canal was labeled as “oriented in occlusal direction.” The opposite was labeled as “oriented in apical direction.” If the position of the accessory canal did not deviate more than 5° from a horizontal level according to the longitudinal axis of the tooth, usually obtained in very short canals (<300 μm), the accessory canal was labeled as “perpendicular.”

The diameter of each accessory canal was measured at its smallest diameter of its narrowest point using a caliper tool in VGStudioMAX version 2023.1 with a precision of 10 μm based on their sub-voxel surface determination. Canals with a diameter smaller than 40 μm could not be measured accurately due to the 40 μm resolution of the microCT data and were labeled as “less than 40 μm.” Patency of the canals was visually checked in VG Studio version 2023.1. When at any point along the accessory canal’s length the opacity of the canal was the same as the surrounding dental material it was labeled as “obliterated.”

### 2.3 Histology

Histological examination was performed on a subset of 3 cheek teeth in which the largest concentration of accessory canals was found during microCT evaluation. Zones for histological examination were selected from each tooth individually based on results from microCT imaging. The teeth were sectioned using a diamond-coated, water-cooled micro-band saw (MBS 240/E, Proxxon S.A., Wecker, Luxembourg) and cutting surfaces were photographed using a stereo microscope (ZEISS Stemi 2000-C, Carl Zeiss Jena, Germany). Subsequently samples were decalcified using buffered EDTA solution (ethylenediaminetetraacetate, pH 8, 20°C), followed by routine processing and embedding in paraffin wax. Sections were cut 7 μm thick and a Toluidine blue staining was performed. The decalcified sections were microscopically assessed for the presence of cellular content, patency and diameter of accessory canals. Finally, the histological and microCT findings were compared.

### 2.4 Statistical analysis

Descriptive statistics for all data were calculated in Excel. Possible correlation between age and number of accessory canals was explored using Spearman’s rank correlation coefficient after checking the data for normality with the D’Agostino-Pearson test using manual calculations in Excel.

## 3 Results

### 3.1 Samples

Micro-computed tomography images were obtained from 15 maxillary and 19 mandibular cheek teeth ranging from Triadan 06 to 11 positions with a dental age between 4 and 9 years.

### 3.2 Micro computed tomography

#### 3.2.1 Number of accessory canals

The total number of accessory canals found in this study was 487 with 339 canals found in maxillary and 148 canals in mandibular cheek teeth. One or more accessory canals were identified in 100% of the maxillary cheek teeth (15/15) and 89% of the mandibular cheek teeth (17/19) examined in this study. The two teeth without any accessory canals were a Triadan 407 and a Triadan 307 collected from different cadavers and both with a dental age of 4 years. The number of accessory canals per tooth ranged from 11 to 44 in maxillary (mean: 22.6 ± 9) and from 0 to 19 in mandibular CT (mean: 7.8 ± 5.9) respectively.

A correlation between dental age and number of accessory canals per tooth was explored using Spearman’s rank correlation coefficient which showed a strong positive correlation (Rs = 0.81, *p* = 7.35*10–9, two tailed test) between these two parameters. Thus the null hypothesis that there is no correlation between dental age and accessory canals per tooth could be discarded with a probability of >99%.

#### 3.2.2 Categorization of accessory canals

**Figure 1:**
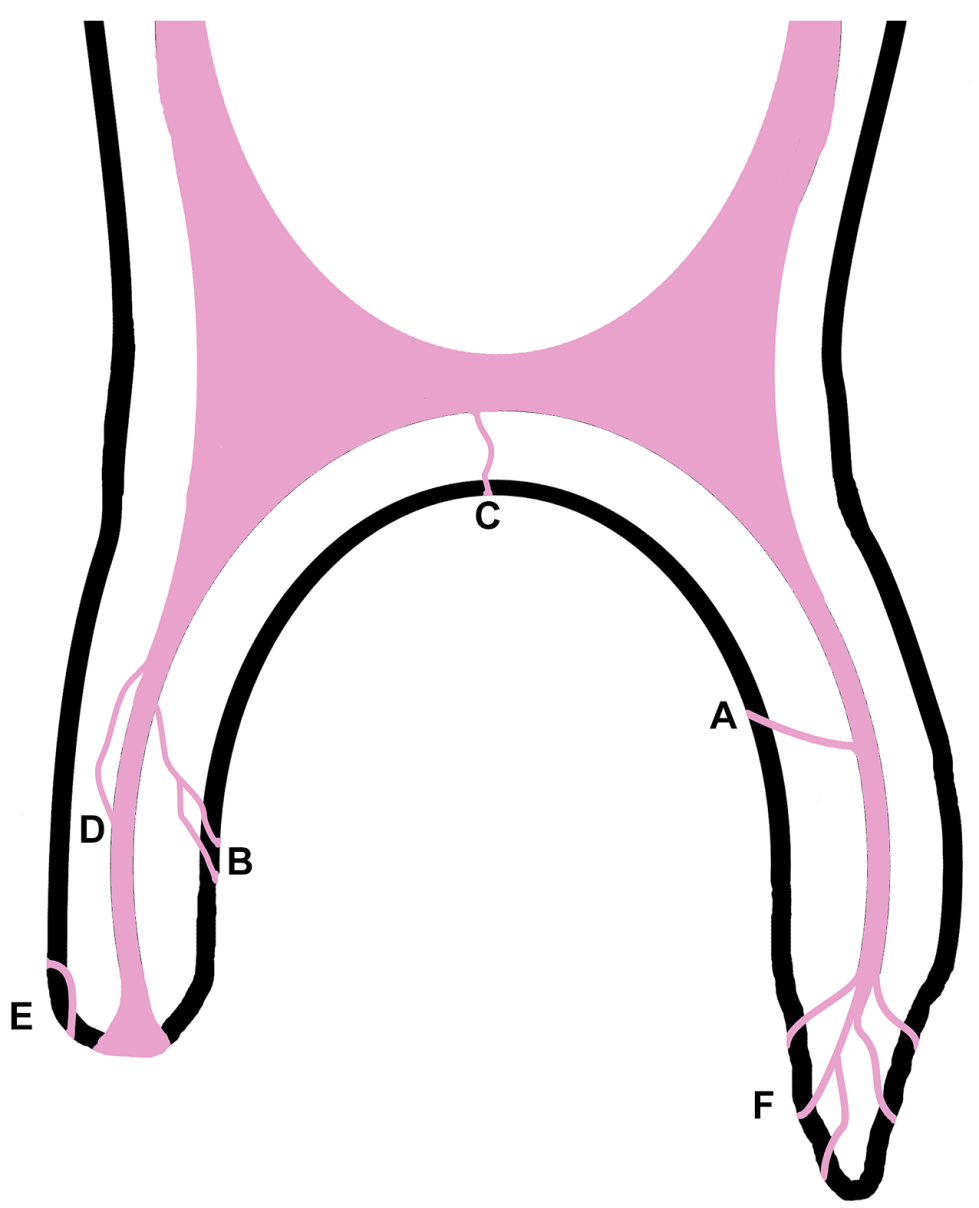
Simplified illustration of the types of accessory canals found in equine cheek teeth: singular accessory canal **(A)**, branching accessory canal **(B)**, furcation canal **(C)**, recurrent canal **(D)**, peripheralic canal **(E)** and apical delta **(F)**.

Several different canal types were distinguished in the cheek teeth examined including singular, branching, furcation, recurrent, peripheral and apical delta as illustrated in Figure 1. 463 (95%) accessory canals were singular (1A) while 24 (5%) exhibited branching at some point along their trajectory (1B). In 4 cheek teeth a single furcation canal was found originating from the floor of the common pulp chamber and exiting at the furcation (1C). In this study this type of canal was found exclusively in maxillary cheek teeth. 11 accessory canals were recurrent (1D), leaving the main root canal and rejoining more apically (1D). 10 accessory canals were peripheral, moving through mineralized dental tissue but without any communication to the pulp system (1E). These canals were usually found near the apex (9/10) and on one occasion in the middle third of the root. Finally, accessory canals could be seen forming an apical delta (1F). The distribution of the types of canals between maxillary and mandibular cheek teeth is presented in Table 1.

**Table 1:** Distribution of the accessory canal types between maxillary and mandibular cheek teeth.

	Singular	Branching	Furcation	Recurrent	Peripheral
Maxillary	326 (96%)	13 (4%)	4 (1%)	7 (2%)	6 (2%)
Mandibular	137 (93%)	11 (7%)	0 (0%)	4 (3%)	4 (3%)

Data presented as number (%).

**Figure 2:**
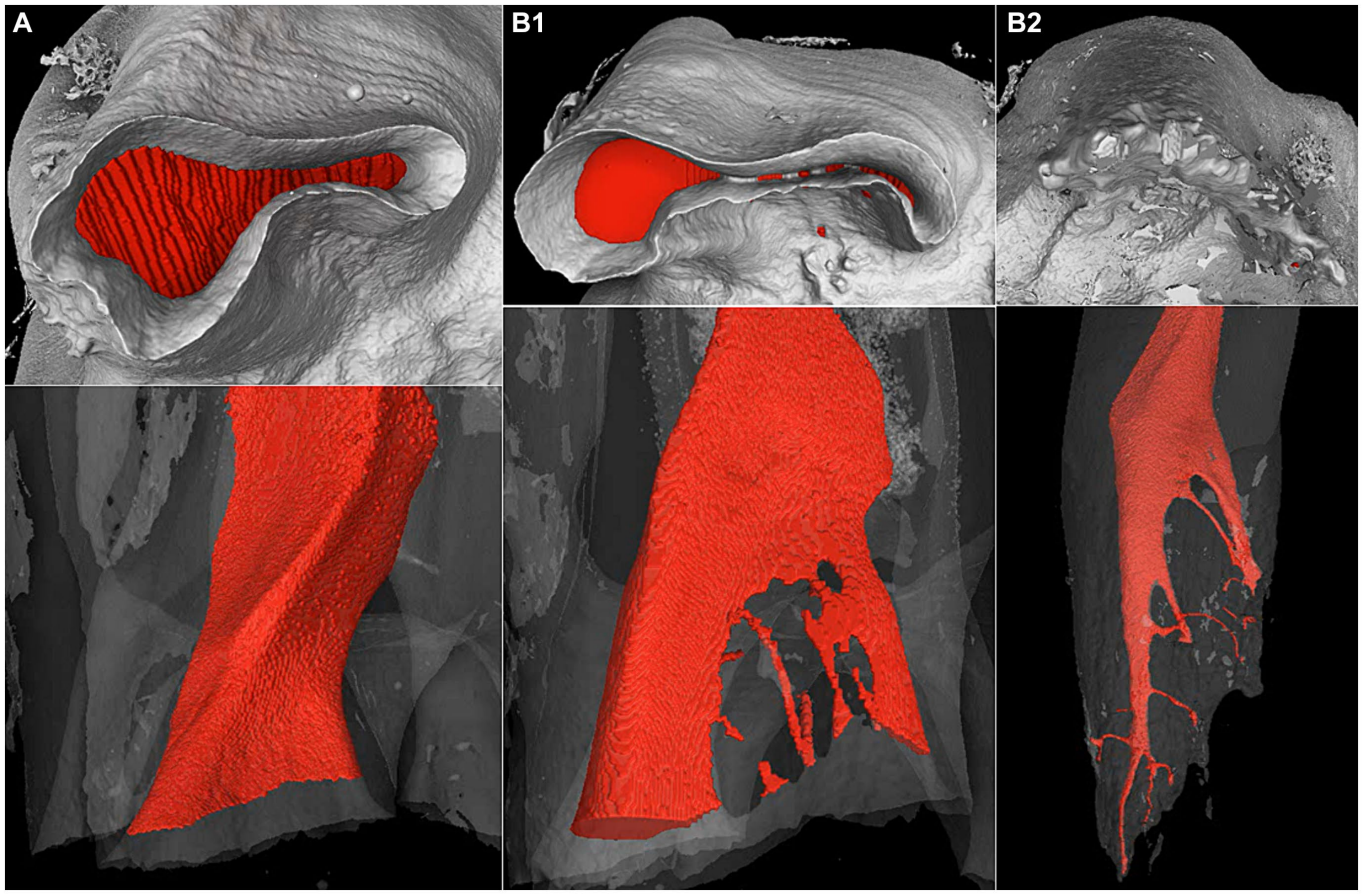
Apical and side view of the three types **(A,B1,B2)** of apical root canal terminations found in equine cheek teeth on microCT images. The pulp cavity has been digitally highlighted in red. **(A)** No apical delta on the mesial root of a Triadan 210 with 7 years eruption age, **(B1)** early stage apical delta on the distal root of the same tooth and **(B2)** mature apical delta on the distal root of a Triadan 106 with 9 years eruption age.

An apical delta, as defined by the AAE glossary of endodontic terms (20) and illustrated in Figures 2B1,B2, was found in 84% of the examined roots. An apical delta was not seen in 16% of examined roots in this study. These roots contained a root canal that ended apically in a large and wide apical foramen without any branching as illustrated in Figure 2A.

Apical deltas could be observed in different stages of development. Early stages were recorded in 72% of examined roots and were characterized by the original central root canal being split up into large segments sometimes combined with accessory canals. The contours of the large apical foramen are still present (Figure 2B1). In 12% of examined roots a mature apical delta structure was observed. A mature apical delta is characterized by an elongated and pointy appearance of root canal without a large apical foramen. This root canal has split up into several small ramifications leaving the apex in different directions (Figure 2B2).

**Figure 3:**
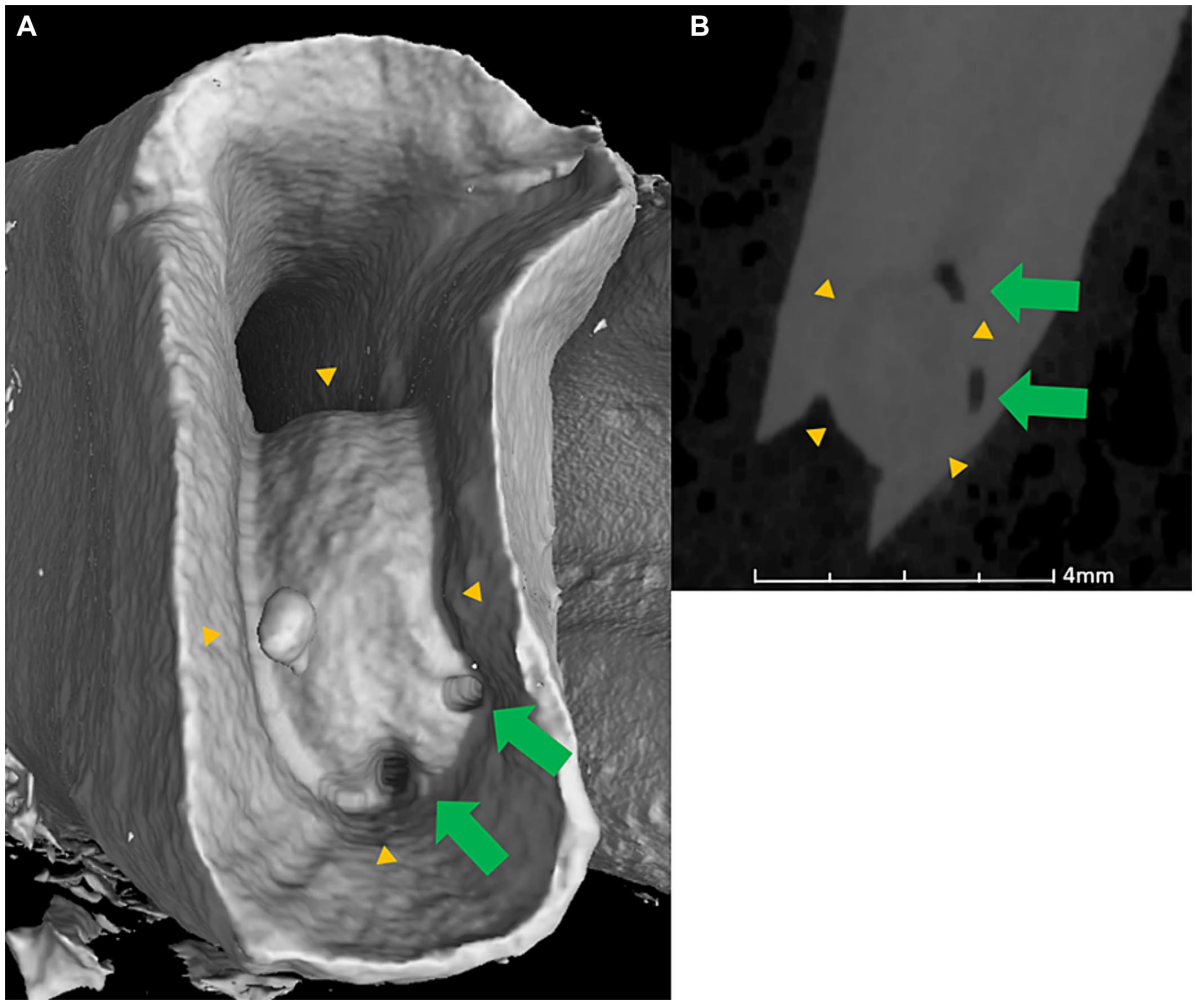
MicroCT apical **(A)** and lateral transverse view **(B)** of the mesial root of a Triadan 409 with dental age of 6 years. Structure resembling a pulp stone (yellow arrowheads) with two patent accessory canals (green arrows) curving around it.

Out of all accessory canals mentioned above, 7 were seen curving around structures resembling pulp stones in the apical third of the roots as seen in Figure 3. These structures showed continuity with surrounding dentine and had a rounded, slightly heterogeneous opacity on the microCT images.

#### 3.2.3 Localization of accessory canals

The localization of accessory canals varied considerably between the roots and along the length of the roots.

Almost half of the recorded accessory canals in maxillary cheek teeth were seen in the palatal root. The presence of accessory canals in mesial or distal roots was comparable for both maxillary and mandibular teeth. The distribution of accessory canals between dental roots is recorded in Table 2.

**Table 2:** Distribution of the accessory canals between the roots of maxillary and mandibular cheek teeth.

	Mesial root	Distal root	Palatal root	Total
Maxillary CT	82 (24%)	93 (28%)	164 (48%)	339 (100%)
Mandibular CT	81 (56%)	67 (44%)	n.a.	148 (100%)

Data presented as number (%).

When examining the position of accessory canals along the roots no accessory canal was found above the level of the common pulp chamber along the reserve or clinical crown. In maxillary cheek teeth the majority (80%, 271/339) of accessory canals were located in the apical third of the root whereas fewer numbers were recorded in the middle third and coronal third of dental roots. The same distribution was observed in mandibular cheek teeth. Distribution of accessory canals along the length of dental roots is illustrated in Table 3.

**Table 3:** Distribution of the accessory canals along the length of the roots of maxillary and mandibular cheek teeth.

Location along the root	Maxillary CT	Mandibular CT
Coronal	16 (5%)	10 (7%)
Middle	52 (15%)	25 (17%)
Apical	271 (80%)	113 (76%)
Total	339 (100%)	148 (100%)

Data presented as number (%).

**Figure 4:**
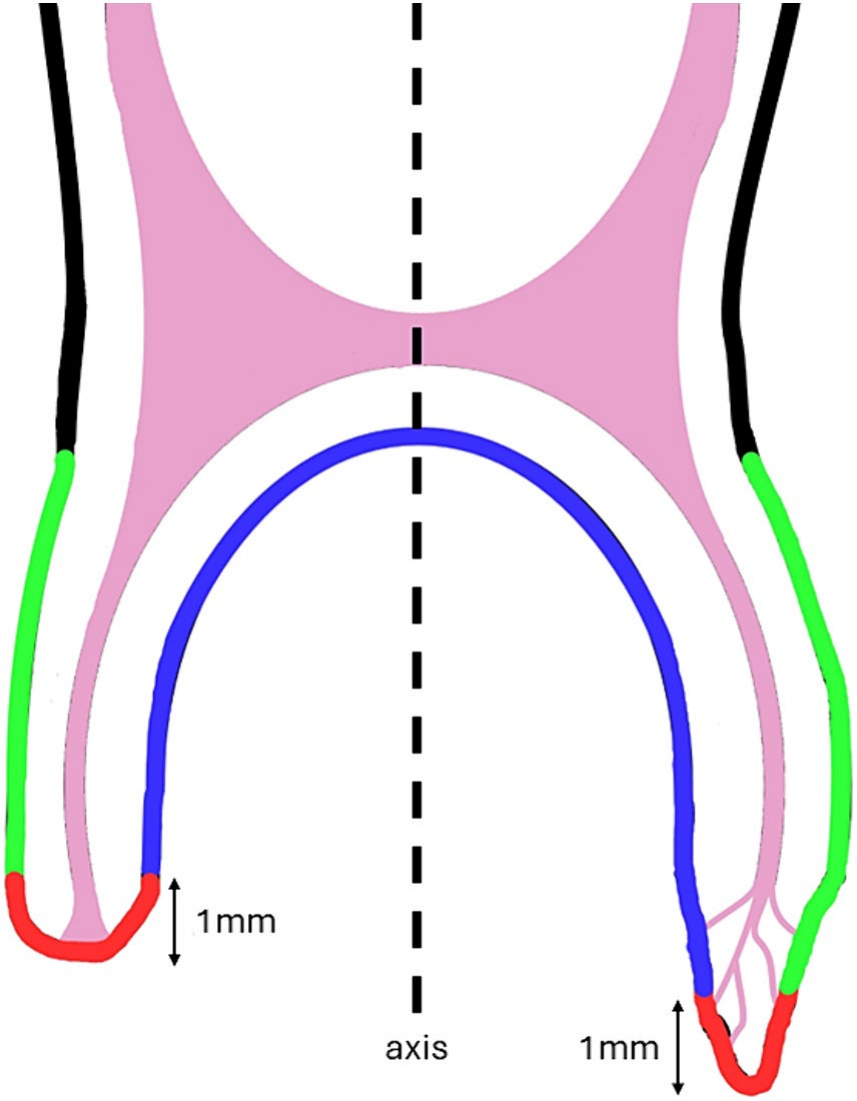
Simplified illustration of accessory canal location along the abaxial wall of the root (green), at the most apical 1 mm (red) and on the axial wall of the root (blue) in relation to the tooth axis on a sagittal cross section.

The accessory canal locations were also recorded relative to the longitudinal axis of the tooth as illustrated in Figure 4.

In the maxillary cheek teeth examined, only 2% (6/339) of the accessory canals were found along the abaxial wall of the root, 46% (155/339), at the root tip (i.e., the most apical 1 mm of the root) and 52% (178/339) along the axial wall of the root.

In the mandibular cheek teeth, 5% (7/148) of the accessory canals were found along the abaxial wall of the root, 38% (57/148) at the root tip and 57% (84/148) along the axial wall of the root.

#### 3.2.4 Orientation of accessory canals

The accessory canal trajectory in relation to the longitudinal axis of the root canal varied considerably among individual teeth and roots. In the majority of both maxillary (63%; 214/339) and mandibular (60%, 88/148) dental roots, the accessory canal trajectory was apically oriented. An occlusal trajectory was recorded in 22% (72/339) and 33% (49/148) maxillary and mandibular cheek teeth roots, respectively. In a minority of cases, the orientation of accessory canals was perpendicular to the long axis of the root canal 16% (53/339) in maxillary and 7% (11/148) in mandibular cheek teeth.

#### 3.2.5 Accessory canal diameter and patency

Accessory canal diameters were highly variable both along their lengths and between canals, but were always smaller than the diameters of the root canals they originated from. In maxillary cheek teeth accessory canal diameters measured on microCT images ranged from less than 40 μm to 770 μm with a median of 120 μm and a mean of 144 μm (±129 μm). In mandibular cheek teeth the diameters ranged from less than 40 μm to 1,150 μm with a median of 100 μm and a mean of 140 μm (±99 μm). Considering the voxel size of 40 μm the difference between the two medians and means is negligibly small.

Based on the microCT images, 93% (316/339) of maxillary and 94% (139/148) of mandibular accessory canals were classified as patent.

### 3.3 Histological analysis of accessory canals

**Figure 5:**
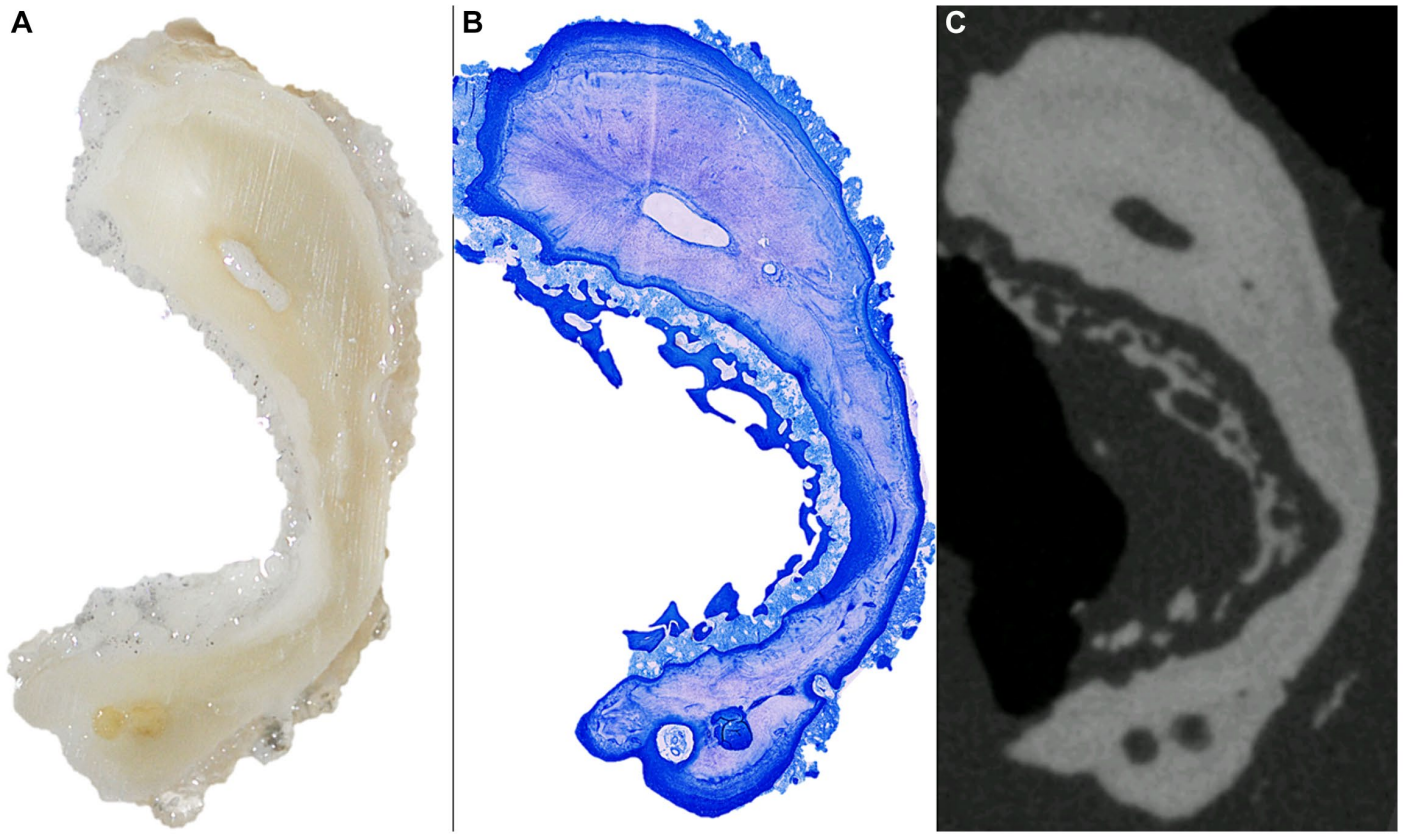
From left to right stereomicroscopical image **(A)**, histological **(B)** and microCT **(C)** cross section of the distal root of a 406 with an eruption age of 9.5 years.

Histological morphology of the root cross sections considerably resembled the cross sections obtained through microCT (Figure 5).

**Figure 6:**
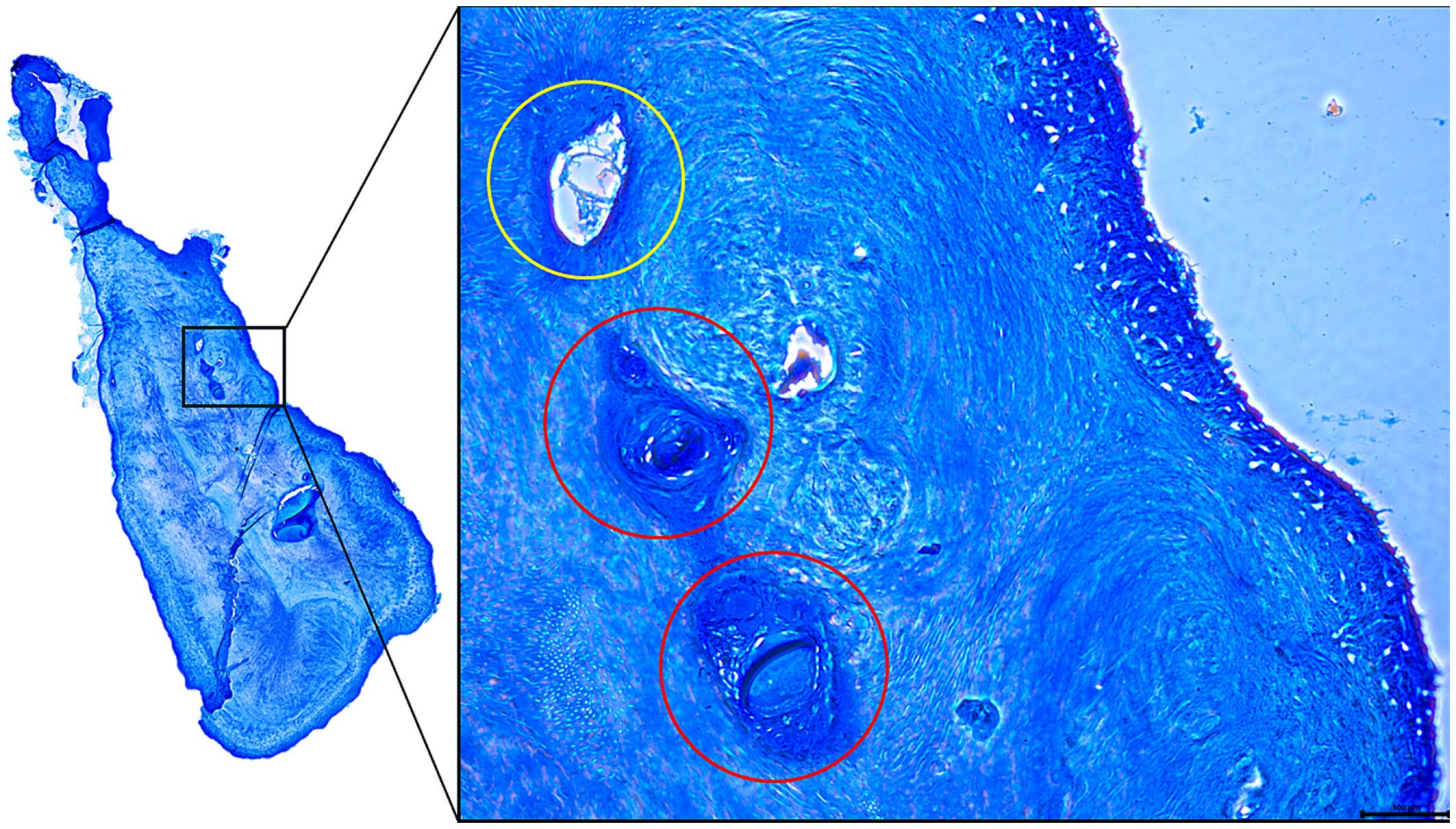
Histological cross section of the mesial root of a 106 presenting several accessory canals. The accessory canal circled in yellow is lined by secondary dentine while the accessory canals circled in red are lined (and obliterated) by porous reparative dentine.

**Figure 7:**
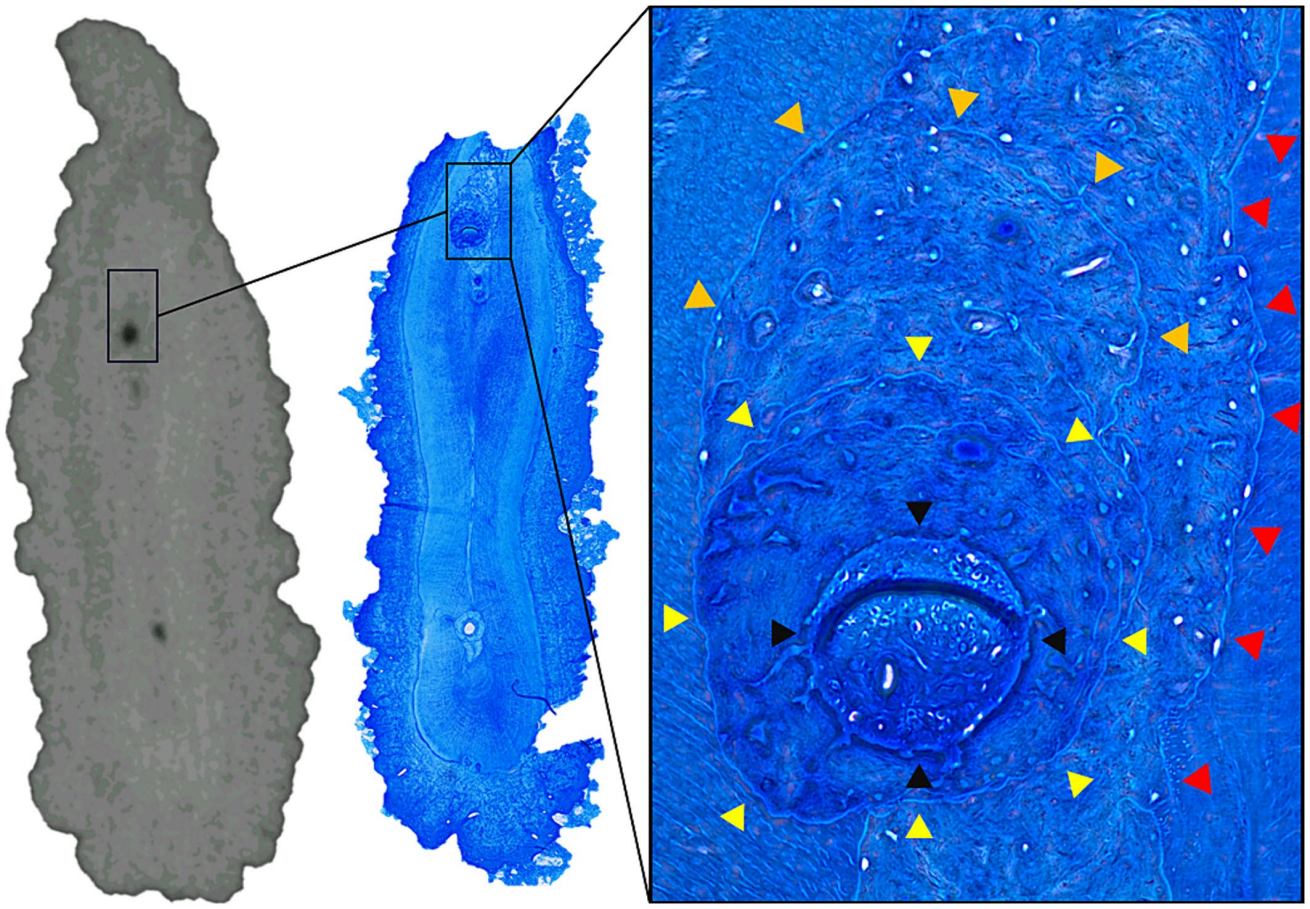
MicroCT and histological slide of the palatal root of a 206 presenting several accessory canals. Note the layered deposition of porous tertiary dentine around and inside the obliterated accessory canal (red, orange and yellow arrowheads accentuating different layers). A clear outline of the accessory canal is still present (black arrowheads).

All accessory canals detected by microCT analysis were also identified in histological sections. In addition to the 19 accessory canals identified in microCT and histological cross sections, 37 additional small, well delineated cross sections of hollow structures were detected by histological examination. All canals were surrounded by either regular dentine, layers of reparative dentine or a combination of both (Figures 6, 7).

**Figure 8:**
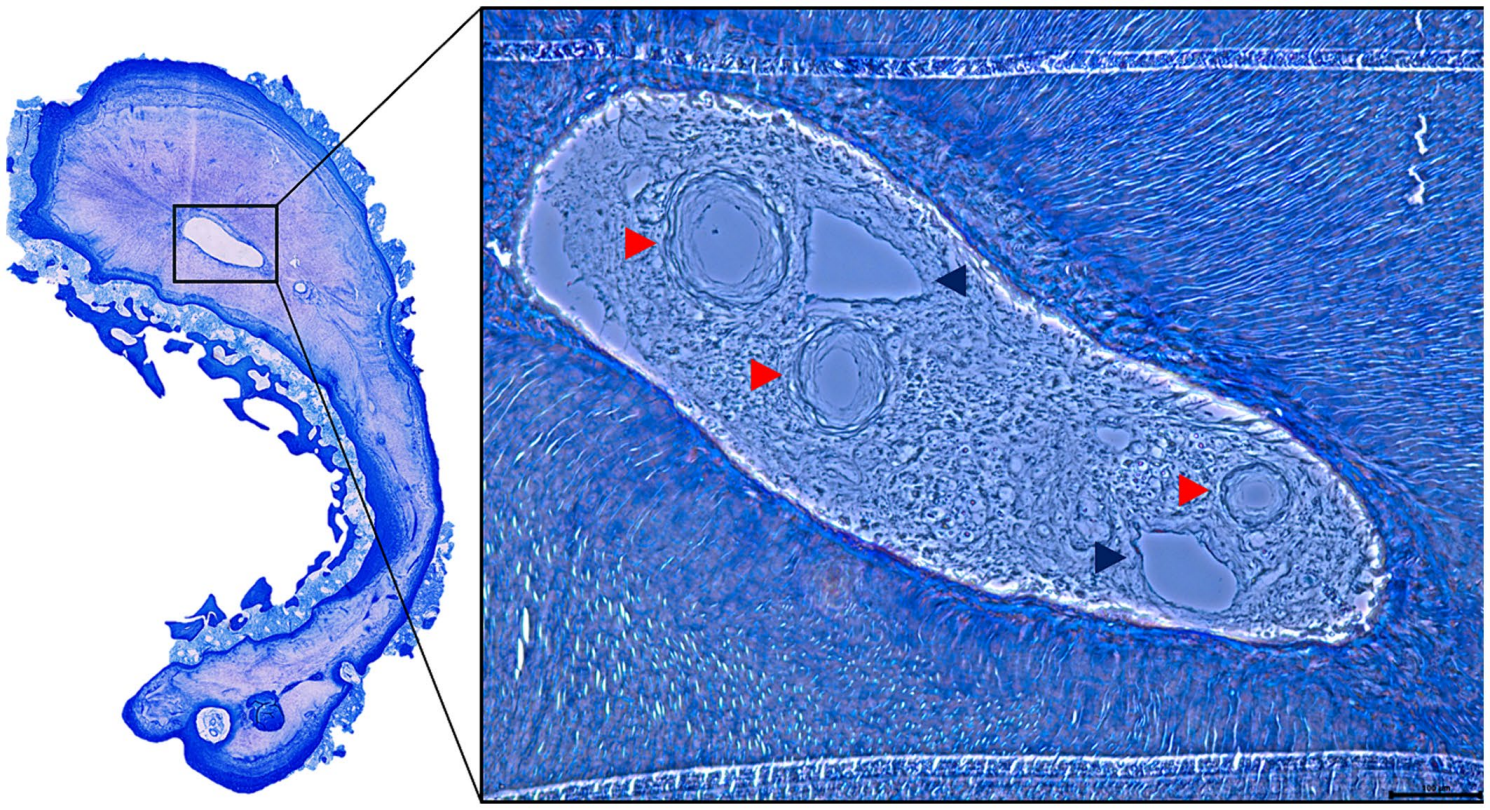
Histological slide of the distal root of a 406 presenting several accessory canals. The lumen contains structures that were recognized as arteries (red arrowheads) and veins (blue arrowheads).

Although a detailed analysis of the cell types inside the canals was not possible, clear outlines of veins and arteries were identified inside some of the patent canals (Figure 8). In 10 of the 19 accessory canals and root canals previously identified as patent based on microCT imaging, a partial or total obliteration of the canal was found on histology. The material obliterating these canals was identified as reparative dentin. A clear outline of the previously patent canal could always be identified on histology. On re-evaluation of the microCT images, a slight increase of radio-opacity could be seen locally inside some of the canals at the level of these obliterations. In 9 out of 10 obliterated canals the obliteration was only partial at the level of the histological section with small parts of the canal still showing patency (Figure 7).

When comparing the diameter measurements of 29 (accessory) canals between matched microCT images and histological slides the differences between the paired measurements ranged from −71 μm to 91 μm with a mean of 12.17 μm and standard deviation of 37.47 μm which is close to the voxel size of 40 μm.

In 10 paired measurements there was an under-estimation of the canal diameter based on microCT images and in 18 paired measurements an over-estimation was detected. In one paired measurement canal diameter perfectly matched between the microCT cross section and histology.

On all roots of all 3 seemingly healthy cheek teeth examined histologically, varying peripheral resorptive lesions could be observed along the abaxial wall of the root combined with the formation of reparative cement.

## 4 Discussion

This study showed that the presence of accessory canals is a characteristic feature of dental roots of equine cheek teeth (prevalence up to 97%). Furthermore, it was shown that the appearance of accessory canals increases with dental age, and thus can be regarded as a result of prolonged root formation and ongoing secondary dentin production within the pulp cavity. Accessory canals provide and maintain vascularization of the dental pulp in the ever-narrowing pulp system of horses. An important degree of individual variability was seen within and in between teeth with respect to orientation, location, diameter and direction.

In humans the incidence of accessory canals for all teeth combined varies between 27.4 and 59% depending on the studied population (18, 37–39). In dogs the incidence of accessory canals not belonging to apical deltas was as low as 2.4% according to one study (24). Opposed to equine cheek teeth, a correlation between dental age and number of accessory canals was not detected in humans (40). As accessory canals are formed during root formation and root formation progresses with increasing age, the positive correlation between these two values seems logical. The fact that the same correlation was not found in human teeth could be due to the large differences in the development of brachydont and hypsodont dentition.

Six types of accessory canals were described in the present study including singular, branching, furcation, recurrent, peripheral and apical delta. The low number (4.9%) of branching accessory canals in equine cheek teeth is similar to the percentage found in humans (8.5%) (18). Such a similarity in percentages was also found in recurrent canals which accounted for 2.3% of all accessory canals in this study compared to 0–2% in human teeth according to Mazzi-Chaves et al. (19). A larger inter-species variation was found in the number of furcation canals which were found to be present in up to 46% of human teeth (15), 27.2% of cat teeth (28), 12% of equine cheek teeth in the current study and surprisingly 0% of dog teeth (24). In our samples, furcation canals were only detected in maxillary cheek teeth while in cats no significant difference was found between maxillary and mandibular cheek teeth (28). The absence of furcation canals in the mandibular teeth in this study can be explained by the fact that the presence of a common pulp chamber in these teeth has only been seen up to a dental age of 2 years (4) while the post eruption age range for this study was 4–9 years. In comparison, a common pulp chamber in equine maxillary cheek teeth could be found up until an age of 6 years post-eruption (4). The term “peripheral accessory canal” was introduced to describe canals that enter and leave the root walls without a direct communication with the dental pulp. However due to this lack of connection to the dental pulp these are not accessory canals as defined by the AAE glossary of endodontic terms (20).

An apical delta was present in up to 100% of permanent teeth of dogs (22, 25) as young as 6 months old (26). These results are in a line with the results of the current study where 32 out of 34 (94%) equine cheek teeth showed apical deltas in different developmental stages and only 2 mandibular cheek teeth with dental age of 4 years presented no apical delta. The anatomy and complexity of equine apical deltas varied greatly compared to the “standard” sprinkler rose anatomy in dog teeth.

In maxillary cheek teeth the palatal roots accounted for 48% of all maxillary accessory canals while the mesial and distal roots accounted for 24 and 28%. This discrepancy can most likely be attributed to the broader anatomy of the palatal root which results in a longer and narrower apical foramen compared to its mesial and distal counterparts. The authors suggest that the age-related physiological deposition of secondary dentine in such a long and narrow apical foramen would likely cause it to segment sooner and into more apical accessory canals than in the other two roots with wider apical foramina. In mandibular cheek teeth the distribution between mesial and distal roots was comparable. A comparison with other species was not possible due to the largely different and highly varying anatomy of their dental roots compared to horses. Nonetheless, a variation in the distribution of accessory canals between individual roots of the same tooth was also found in dogs (25).

The present study showed that the prevalence of equine accessory canals increased along the root from occlusal to apical, i.e., occlusal or furcation (6%), middle (16%) and apical thirds (78%). A similar distribution pattern has been seen in human teeth, with 0.7% of the detected accessory canals in the occlusal third of the root or at furcation level, 12.8% in the middle third and the majority of 86.5% in the apical third (18). One might speculate that as the cheek teeth age, continuously erupt and the roots elongate the previously apical accessory canals slowly narrow like the rest of the pulp system does until they are completely obliterated. In the meantime, new accessory canal formation takes place at the apex which might explain these numbers. Furthermore, when root elongation ceases and therefore no new accessory canals are created, the authors speculate that all accessory canals will slowly obliterate until the tooth is lost. Thus, the positive correlation between increasing dental age and increasing number of accessory canals seen in the limited age range of this study might turn into a negative correlation in geriatric horses. The mostly apical and axial localization of accessory canals might be suggestive of a vascular pattern that originates primarily axially and apically from the tooth resulting in a higher concentration of entrapped vascular canals on the axial face and tip of the roots after root mineralization.

Studies on other species measuring the diameter of accessory canals showed similar results to the present study in which a mean diameter of 142 μm was found. Mean diameter of furcation canals in cats was 104 μm (28). In dogs, apical foramina sizes varied between 20 μm and 150 μm (41). In human teeth, accessory canals had a median diameter of 132.3 μm according to one study (17).

In the subset of teeth that also underwent histological examination the difference between the canal diameters measured on microCT and histological images differed in almost all cases with both over and under estimations. This could be explained based on slight imperfections in matching the microCT and histological cross sections. In addition, the microCT images have a relatively low resolution compared to histology. This produces slight visual blurring of the canal walls inducing inaccuracy during measurement of the canal diameter.

However, when it comes to canal obliteration, a discrepancy was found between microCT interpretations and histological findings with more obliterated canals on histology than what was seen on microCT images. This can be explained by the often porous “osteodentin” found in many of the obliterated canals which, due to its porosity, produced a darker shade of gray on microCT images than the surrounding dentin creating the impression of patency. Despite the fact that some accessory canals were found locally obliterated by tertiary dentine on histology, small openings could be found in most of these obliterations. The lumen of these small openings was always larger than that of the dentinal tubules. These findings imply that the obliterations seen on microCT in such canals do not necessarily account for a perfect seal in every case. Additionally, it is also expected that in healthy cheek teeth, some of these canals are not obliterated as long as there is vital pulp inside the tooth. This has been shown to be the case at least until the age of 27 years in teeth which showed no macroscopically visible apical foramen but vital pulpal tissue within the tooth (2).

Interestingly, in all of the investigated cheek teeth, minor peripheral root resorption and reparative cementum formation was noticed. These cheek teeth showed no macroscopical signs of dental disease but had a dental age > 9 years and therefore showed relatively short reserve crowns and long delicate roots which have to dissipate the occlusally applied masticatory forces. As it is known that excessive loads can trigger resorptive processes – which in turn can become repaired by cementoblastic reactions under non-infectious conditions – it appears most likely that the recorded resorptions represented an age related adaption to changed biomechanical conditions rather than a dental pathology. The presence of vascular structures inside accessory canals was shown in this study. Unfortunately, a more detailed analysis of the cellular content of accessory canals was made impossible by the long storage of these samples in a 0.5% chloramine solution.

The intricate system of accessory canals providing additional blood supply in healthy teeth have been shown to become a safe space for biofilms and bacteria in case of pulp infections in humans. This is a known cause of failure of endodontic therapy in the human dentition (30–32). The aim of an endodontic treatment in the first place is cleaning and disinfection of the pulp system. This is usually done using direct debridement methods where various rigid instruments are introduced into the pulp cavity system in combination with indirect debridement using various fluids and creams such as saline, concentrated NaOCl, EDTA, etc. (42). Due to the variable patency, direction, location and small diameter of the accessory canals seen in this study, successful mechanical debridement of these canals using rigid instruments is highly unlikely. Thus, in case of an orthograde endodontic treatment the clinician is left with indirect methods using various fluids to attempt cleaning and disinfection of these complexities. In human literature heating, ultrasonic and laser activation of these fluids are suggested to improve the penetration and efficacy of these fluids inside accessory canals. After cleaning and disinfection, the use of pulp medication such as MTA and CaOH is also advocated to further disinfect the pulp cavity (30, 33). In severe cases apex resection has been suggested as a treatment option (43, 44).

Opposed to humans, in horses the clinical relevance of accessory canals remains to be demonstrated. The presence of tertiary dentine in some equine accessory canals in this study suggests that these canals are armed with the same defense mechanism against apical infection as the rest of the pulp system. Therefore, these accessory canals might get obliterated by tertiary dentine in case of infection, which in turn might minimize colonization of and persistence in these canals by bacteria in horses. On the other hand, the porosity of the dentine inside some of the accessory canals identified in this study and the only partial obliteration of other canals might still allow an important level of bacterial colonization. Visualization of the presence of accessory canals in horses in a clinical setting is impossible at this time but due to the high prevalence seen in this study their presence should always be assumed.

For this reason caution is advised when performing endodontic treatments in horses. The use of the precautionary measures described above can be useful to mitigate the chances of failure of treatment. The efficacy of these techniques in horses requires further investigation.

To conclude, accessory canals are very common in equine cheek teeth and show a high intradental and interdental variability. Their role in pulp disease and persistent pulpal infection deserves further research. At the same time, experiments are required in order to improve the effectiveness of endodontic disinfection in equine endodontic therapies.

## Data availability statement

The raw data supporting the conclusions of this article will be made available by the authors, without undue reservation.

## Ethics statement

Ethical approval was not required for the study involving animals in accordance with the local legislation and institutional requirements because samples were collected post-mortem from horses that either died or were euthanised for reasons unrelated to the study. Owner consent for the use of these samples was acquired at the time of admission of the patient.

## Author contributions

SK: Conceptualization, Formal analysis, Investigation, Methodology, Validation, Visualization, Writing – original draft, Writing – review & editing. CS: Writing – review & editing, Visualization, Validation, Supervision, Software, Methodology, Investigation. MB: Investigation, Resources, Software, Visualization, Writing – review & editing. IJ: Writing – review & editing, Visualization. JV: Visualization, Investigation, Writing – review & editing. LV: Project administration, Resources, Supervision, Writing – review & editing, Funding acquisition, Conceptualization.
